# An Explorative Study of Qualities in Interactive Processes with Children and Their Parents in Music Therapy during and after Pediatric Hematopoietic Stem Cell Transplantation

**DOI:** 10.3390/medicines6010028

**Published:** 2019-02-18

**Authors:** Lena Uggla, Katarina Mårtenson Blom, Lars Ole Bonde, Britt Gustafsson, Björn Wrangsjö

**Affiliations:** 1 Astrid Lindgren Children’s Hospital, Karolinska University Hospital-Huddinge, SE 141 86 Stockholm, Sweden; 2 Cell Therapy and Allogeneic Stem Cell Transplantation, Karolinska University Hospital-Huddinge, SE 141 86 Stockholm, Sweden; 3 Department of Clinical Science, Intervention and Technology, CLINTEC, Karolinska Institutet, SE 171 77 Stockholm, Sweden; britt.gustafsson@ki.se; 4 Psychotherapy, Supervision and Consultation in Private Practice, Mentibus AB, Mössebergsvägen 32, SE 167 43 Bromma, Sweden; kia.m-blom@telia.com; 5 Department of Communication and Psychology, Aalborg University Copenhagen, A.C. Meyers Vaenge 15, DK-2450 Copenhagen SV, Denmark; lobo@hum.aau.dk; 6 Samklang Konsult, Garvargatan 17, SE 112 21 Stockholm, Sweden; bjorn.wrangsjo@gmail.com

**Keywords:** music therapy, children, pediatric, families, hematopoietic stem cell transplantation, cancer, collaborative research interview

## Abstract

**Background:** Hematopoietic stem cell transplantation (HSCT) is an established treatment for severe disorders of the pediatric hematopoietic system. However, there is a need for supportive interventions due to physiological and psychological strain. Music therapy is used in health care to help patients through difficult experiences and enable well-being. Our previous randomized studies showed significantly reduced heart rates four to eight hours after intervention as well as increased health-related quality of life. **Methods:** The aim of this qualitative study was to explore the participants’ and parents’ own experiences of the interactive processes during the music therapy intervention. Six families were included. The data collection used collaborative research interviews. An independent psychologist facilitated the interviews with the children, the parents, and the music therapist and also performed the analysis. **Results:** Three main themes emerged: experiences of competency and recognition of self, interactive affect regulation as change potential, and importance of the therapeutic relationship. **Conclusions:** For the participants, music therapy developed into a significant and helpful experience, an important ingredient in coping with and managing the treatment period at the hospital.

## 1. Introduction

Pediatric hematopoietic stem cell transplantation (HSCT) is an established treatment for specific malignant and nonmalignant hematopoietic diseases [1]. Medical progress in HSCT procedures has led to increased HSCTs, and overall survival has improved in the pediatric group [1,2]. The HSCT procedure is very intense, especially for the first three to six months, with a long period of isolation, mainly due to the risk of infection.

After the primary hospitalization, the child is monitored frequently, to allow early detection of, e.g., graft-versus-host disease (GVHD), infections, or possible relapse of the disease. The treatment has a major impact on the child’s and the parents’ psychological status, the family’s inner relations, and the health-related quality of life (HRQoL) of the child and parents [3]. The lowest ratings of the child’s HRQoL are noted one and three months after the HSCT [4]. It takes approximately one to three years to return to the same level of HRQoL as before HSCT [3,5]. The relationship between the HRQoL of the child and the parents’ emotional functioning in the HSCT context is complex [6]. Risk factors for impaired HRQoL for the child are female gender, GVHD, older age, and receiving stem cells from an unknown donor [3,5,7]. 

Going through HSCT is potentially life-threatening, and children are often aware of the severity of their disease and the treatment. Among numerous psychological reactions, posttraumatic stress disorder (PTSD) and traumatic stress symptoms have been reported in HSCT survivors [8], as well as neurocognitive dysfunctions [9]. Trauma can create serious disturbances in the integration of cognitive, sensory, and emotional processing [10]. If the impact of the trauma remains unsolved, symptoms of PTSD like emotional numbness, hyperarousal, intrusions, and avoidance (described in DSM-5) may occur [11]. Going through HSCT exposes the child and the family members to the risk of moving outside their “window of tolerance” [12]. This “window” is the individual zone in which one is awake, calm, and safe enough to be able to be curious, creative, social, and learning [12]. Previous research reported high levels of stress and depressive symptoms in families of children going through HSCT [13] and parental depression and family functioning interacted with the distress of the child [14]. In families where a sibling is the stem cell donor, the parents experience higher distress and the donor reports poorer HRQoL [15,16], although in one study the donors experienced both positive and negative effects [17]. Riva et al. reported that 11–198 months after HSCT, approximately 15% of parents estimated high levels of distress and 25% reported posttraumatic growth [18].

Emotional regulation is an important component of mental health [19]. In infancy, the parents’ interactions with the child powerfully facilitate the development of emotional regulation [20,21]. Infants are biologically prepared to encounter others in intersubjective meetings with joint attention, sharing intentions and affective states [22], which continues through life. Music affects the whole brain: the left and right hemispheres, the prefrontal cortex, and the brainstem [23]. Music affects different neurochemical systems of reward, motivation, and pleasure, and has the potential to reduce stress and strengthen social connections [23]. 

Both listening to and playing music can have positive effects on different biomarkers, e.g., immunoglobulin and cortisol levels [24,25]. Listening to familiar music, singing, creating, and improvising are suggested to have an impact on emotional regulation and reduce the activity in the amygdala [26,27]. Music evokes and affects emotions [28], and the use of music in emotional regulation is supported by behavioral and neural evidence due to music’s function in early infant–parent bonding and its developmental fitness [29]. The inborn communicative musicality shared by infant and parent is the basis for, e.g., communicating, learning, remembering, and expecting, and later for acquiring language [30]. Music therapy is used in pediatric health care with the goal of helping children through serious experiences and promoting health [31]. Music therapy is a relational therapy, and a main objective is to increase the patient’s experiences and intersubjective knowledge by being involved and relating through music [32,33]. The concept of “musicking” explains music as an interpersonal process instead of an object: a social, culture-related, relational, and interactive process [34,35,36]. Musical interventions have been shown to have a positive impact on children with cancer [37,38]. 

Previous research reported increased well-being and decreased procedural pain [39,40] after music therapy. Children going through HSCT reported reduced levels of anxiety after songwriting [41], and young adults reported courageous coping, social integration, and family environment [42] after therapeutic music video intervention. In our earlier research, children randomized to music therapy during the HSCT procedure were reported to have reduced heart rates four to eight hours after intervention [43], and HRQoL increased at discharge from the hospital at four to six weeks as well as at six-month follow-up after receiving music therapy [44]. We wanted to explore the involved children’s and parents’ own experiences of the interactive process during music therapy through interviews. Qualitative studies of children’s and parents’ experiences are rare in the literature, and it is important to know more about the interaction in music therapy and how it influences the participants in a pediatric setting. 

The aim of this qualitative study was to investigate the subjective experiences and memories of interactions between children, parents, and a music therapist during music therapy interventions. The hypothesis of the study is that it is possible to identify important components and potential common threads in these interactions.

## 2. Materials and Methods 

### 2.1. Previous Randomized Study

From February 2013 to November 2017, a randomized study was conducted at Karolinska University Hospital, Sweden, including 38 patients from 2 months to 17 years of age. Music therapy was performed twice a week for 4–6 weeks in the music therapy group. The patients were hospitalized until donor engraftment. After engraftment, the children were monitored in an outpatient pediatric ward at the hospital. The children in the control group were offered music therapy after discharge in the outpatient ward, twice a week for 4–6 weeks. The randomized study and characteristics of the study population were presented in more detail in a previous study [44].

### 2.2. Music Therapy Protocol in the Randomized Study

The music therapy intervention included both expressive and receptive methods [45]. The session was situated in the child’s hospital room. The child was offered the opportunity to play different musical instruments, sing, and listen to music with the music therapist, and the parents and siblings could also participate. The music therapy setting aimed to build a relationship, a safe therapeutic alliance, between the child and the music therapist [45]. The session had the goal of being flexible, varied, and person-centered [31]. It was designed to provide a holding structure to benefit both children and parents so they could stay emotionally regulated [46] according to theories of affect regulation.

To ensure that the music therapy intervention was held within the child’s window of tolerance, the focus was on supporting the child to be in a here-and-now interaction [47] and to take initiative. This could involve listening to or singing familiar/preferred music, exploring different instruments, improvising, and/or creating songs. The session could also include moving to music/dancing or painting to music, all adjusting to the child’s physical and psychological status [31]. Musical effects can trigger stress symptoms when in an aroused mood. Therefore, it was important for the therapist to be careful concerning selecting new, unknown music, the volume, the pace, not too many chord changes or other unexpected shifts, as well as music in minor keys [26].

### 2.3. Participants in the Interview Study

The goal was to interview 6 families, 3 from each treatment group, in the randomized music therapy study. A total of 8 families were asked in chronological order to participate in the collaborative research interview together with the music therapist. Two families did not want to participate. Six children were included, 2 girls and 4 boys, 3 from the music therapy group and 3 from the control group. The ages of the children at the time of the interview ranged from 1 to 18 years. The interview took place 7–13 months after HSCT and the duration was 45–60 min. Five interviews were conducted in a music therapy room at the hospital, with the possibility for the children to make drawings during the interview. In the music therapy room, there were a lot of instruments, which enabled the children to take initiative to reconnect with the music, making music together with the therapist. Sometimes everyone in the room did some music together. One interview took place in the child’s hospital room for medical reasons, and some small instruments were brought to the room. This study used a qualitative design with both an inductive and deductive approach [48]. The data collection method was the collaborative research interview [49,50]. This is a qualitative interview format where the professionals’ efforts are in focus as well as feedback from children and parents on the collaborative climate during the meetings. The music therapist participated with the patient and family. An external interviewer conducted the interviews. The interviewer is a licensed psychologist and psychotherapist, supervisor, and trainer in psychotherapy. The interviewer was informed about the music therapy protocol and an interview guide was developed in conjunction with the music therapist to satisfy the purpose of the study. The interviewer’s task and responsibility was to follow the themes of inquiry and pay attention to individual participants’ choices of important themes. The interviewer was independent and had no earlier connection to the music therapy provided to the families. 

Each interview was audiotaped and then analyzed through thematic analysis by the interviewer. The dual role as both interviewer and analyzer can be considered a bias [51], and the consequences are accounted for below.

### 2.4. Thematic Analysis of Audio-Recorded Interviews: Procedure

Six questions were defined in the interview guide: (1) How was it to be involved in music therapy? (2) How was it to play music in this way? (3) What was the best/the worst? (4) Is there anything special you remember from when you played, sang, improvised? (5) How was it to get music therapy during the HSCT/after the HSCT? (6) From the therapist’s perspective: ad hoc questions formulated when meeting the child and the parents. 

The thematic analysis followed a procedure in 3 steps:

1. Open listening through all interviews.

2. Focused listening through one interview at a time, taking notes connected to the 6 questions in the inquiry (Appendix Table A1).

3. Systematically editing the text (see above); analysis with recursive listening to the recordings.

### 2.5. Line of Argument Concerning Role and Task of the Interviewer

Conducting a collaborative interview entails acts and intentions by the interviewer in accordance with the intentions behind the interview format [52]. The format was developed within the method of the “reflecting team” [49] and provides a structure with turn-taking between dialogue and listening positions, reflection and response between interviewer, clients, and therapists, all present in the same room.

There are some fundamental assumptions to support having the interviewer and analyzer of the data be the same person. First, it was assumed that verbal expressions by interviewees are just one layer of information to be registered. The second layer, nonverbal interactions, can be registered through video recordings and through sounds on the audio recordings in combination with memories from the interview situation collected by the interviewer [51]. In this specific study, the latter was preferred for practical reasons and to support the intentions behind the aim of the study. However, the intention behind the analysis was to stay open to the possibility that it could generate surprises or new findings, preferably in the implicit interactions between participants during the interviews. Second, it was assumed that when deliberately setting up interviews with participants and therapist present in the same room, the reunion evokes implicit memories of moments from the experiences and collaborative qualities in the conducted sessions. These recollections/memories and reunions are assumed to contain important information about the quality of the experience. In accordance with this assumption, the interviewer’s task is to both navigate through and memorize the verbal answers to the themes of inquiry and important implicit moments of the meeting. As part of the process, the balance between accuracy in recollecting memories from the interview situation and trustworthiness in conducting the analysis needed a clear line between actual verbal testimony and the interviewer’s registration of memorized interactions evoked through listening to the recordings. This description is based on the interviewer’s experiences of conducting collaborative interviews in clinical settings [53] and research settings [50]. In Appendix Table A2, this line is depicted in the text with actual verbal testimonies in italics and the interviewer’s registration of memorized interactions in ordinary text.

The chosen interview format, in which the music therapist participated in the room, facilitated capturing the interviewees’ memories and implicit, nonverbal interactions. No member checking of the data was conducted due to ethical considerations.

The study was carried out according to the Declaration of Helsinki, and the Regional Ethical Review Board in Stockholm, Sweden, approved the study (2015/100-31 and 2017/1845-32). Informed consent was obtained from the parents and from children over 7 years of age. The aim of the study was formulated as follows: to investigate the subjective experiences of interactions between children, parents, and music therapist during the intervention. The chosen questions in the inquiry (Appendix Table A1) were assumed to fulfill this aim. The answers were verbally and nonverbally conveyed during the interviews, and recollected by the interviewer through the analysis.

## 3. Results

### 3.1. Thematic Analysis: Compilation of Results

The experiences were described and conveyed from three perspectives: the child’s, the parents’, and the music therapist’s. The analysis generated an assembled picture of the participants’ experience of each question in the inquiry (Appendix Table A1). Each question constitutes a headline followed by analyzed assumptions (Appendix Table A3). Each assumption is illustrated with excerpts (Appendix Table A2) from the analysis.

### 3.2. From the Analysis

Three themes emerged in the analysis: (1) experience of competency and recognition of self, (2) experience of interactive affect regulation as potential for change, and (3) experience of the importance of the therapeutic relationship.

The following descriptions also contain a development in terms of assumptions, or interpretative analyzing steps toward a final discussion. References were made to relevant research concerning interaction, affect theory, and theory of trauma and dissociation. 

**Figure 1:**
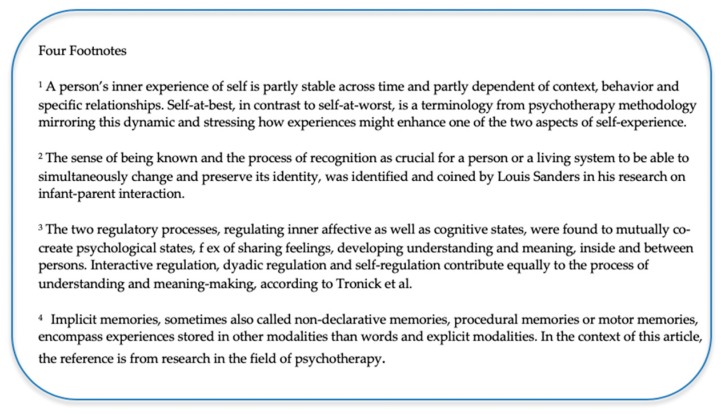
Four footnotes.

1. The participants’ answers showed/conveyed an experience of competency and recognition of self (compiled analysis of answers to Questions 1 and 2). Recollection in the music therapy sessions evoked former experiences of music. Music therapy was experienced as a break in the monotonous isolation and as a preservation of connection to life outside the hospital. Thus, music therapy evoked a sense of being in life, being alive. On the psychological level, music therapy also activated positive emotions or affects that promote curiosity, joy, liveliness, and energy arousal. Sometimes the positive affects helped with diversion from pain, and perhaps fear. On the level of bodily affective experience, music therapy activated bodily sensations of joy, curiosity, and energy arousal, affecting autonomous regulatory systems. Memories of former experiences of competency were evoked, which in the moment nourished inner feeling states of self at best [54] and self-assertion^1^ (Figure 1).

From the perspective of interactive regulation (with the therapist and family members), music therapy generated experiences of recognition^2^ [55,56] and memories of interactive regulation and self-regulation^3^ [57]. Sensorimotor, tactile, auditory, and visual experiences of handling instruments, singing, and moving the body were assumed to evoke implicit memories^4^ [58] of the same or similar behaviors. It is assumed that this entailed feelings of competency, self-regulation, and interactive regulation with self and others, in combination with affective arousal. This, in turn, is assumed to amplify experiences of safety, trust, confirmation, and sense of belonging, all cornerstones in evolutionary rooted motivational systems.

2. The participants’ answers showed/conveyed experiences of interactive affect regulation as potential for change (compiled analysis of answers to Questions 3 and 4). The participants described having felt happy, recognized, emotionally moved, energized, and connected to longing and liveliness in the interactive process. Moments with both a positive and negative experiential flavor entailed the acknowledgment and importance of the relationship with the therapist. This in turn illuminated the importance of the framework of the music therapy, such as number of sessions and feelings of attachment to the music therapist. As a consequence, music therapy evoked positive experiences as well as negative, such as longing for music therapy and the therapist and sometimes the experience of a shortage of sessions. Opening up to interaction and collaboration during music therapy also opened experiences of not getting enough, not having needs met, not being regulated, losing energy. The whole treatment situation could be considered traumatic, therefore putting both the child as an individual and the family as a system into a vulnerable state, outside their window of tolerance [12].

3. The participants’ answers showed/conveyed experiences of the importance of the therapeutic relationship (compiled analysis of answers to Question 6).

The last theme of inquiry that was explored also served as an introduction to each interview. It was intended to facilitate participants’ reflection of the overall process from a meta-perspective. Participants acknowledged the experience of having been recognized, and also their appreciation of the therapist’s contribution to the music therapy. The therapist, from her perspective in remembering the meetings, recalled a kind of narrative about the process of meeting, engaging, and accomplishing the process with each participant. The fifth question: How was it to get music therapy during the HSCT/after the HSCT? was not included in the compilation, since the answers conveyed that every child and family experienced their period to be the most adequate. The question in that sense did not provide adequate information, and the issue might have been explored differently.

### 3.3. Compiled Themes from the Analysis

The following six themes that were compiled are the essence of the analysis on a different abstraction level, formulated to take the discussion further. The themes are all crucial experiential qualities, especially when a person is in a potentially traumatizing situation. The qualities are crucial in both preventing and recovering from traumatizing experiences [59]:

- Being and feeling alive
- Regulation of positive emotions, affects
- Competency, mastering body movements and sensations
- Recognition in meeting the therapist, themselves with family members and the music
- Self at best, experiencing self in relation, self-assertion
- Regulation of safety, trust, and belonging

## 4. Discussion

The aim of this study was to explore children’s and parents’ own experience of the interactive processes during the music therapy intervention. The data collection method, the collaborative research interview, has been commonly used to evaluate therapeutic interventions within different psychiatric and social clinical contexts. It was also used in a study with adults receiving The Bonny Method of Guided Imagery and Music [60] as intervention. This study is the first to use the method in a pediatric setting. Previous research reported PTSD symptoms observed in children going through HSCT as emotional numbness and withdrawal [61]. In another study, 80% of the children showed moderate posttraumatic stress symptoms (PTSS) three months after HSCT [8]. On the contrary, Phipps et al. concluded that there was no evidence for increased PTSD or PTSS in youths with cancer [62], although the study did not report whether HSCT patients belonged to this group. Due to the stressful situation during HSCT for the entire family, the parents may be less receptive to the child’s needs [14]. From the child’s perspective, confidence in the parents is threatened since the child is endangered [20]. The child’s need for support from an “evoked inner companion” [22], built on internalized experiences of being cared for by a self-regulating other, might not be strong enough to regulate the trauma effects. The intervention in the study kept the children in focus, and the results showed the children emerging as individuals taking initiative in the relational process.

**Figure 2:**
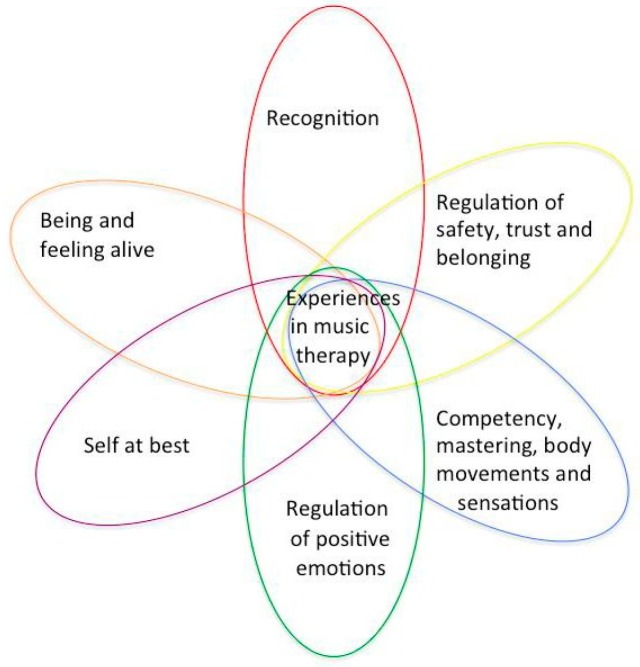
Six main themes from the analysis of responses from children and parents in the study.

The results also displayed the importance of the participation of the parents, as witnesses and helpers to their children. This may have enabled the children to be in the present and be supported in their interaction with the music therapist. The collected results are presented in six themes. The themes, or qualities in the interaction, can be assumed to be linked and concurrently affect one another in a circularly emerging process. The themes are assumed to appear more or less clearly in different sessions, individually and uniquely for each child or parent (Figure 2).

The figure depicts a suggested illustration of the process in the intersubjective field during music therapy. Music experiences in a broader health context (“health musicking”) can be described as “affirmative and corrective, bodily, emotional and relational experiences through musicking” [47].

Intersubjectivity, a concept from theories of developmental psychology and phenomenology, refers to “the field of psychological belonging and seclusion that contains and develops each subject’s sharing of lived experience between humans and other living beings” [63].

The main components in the sharing of lived experience are shared attention, shared intentionality, and shared affectivity [22]. In the intersubjective field, the subject becomes recognized in an interpersonally created and shared world of meaning [22]. The subject’s intention and synchronization with the other’s mind occurs through immediate exchange (regulation) of affects and joint attention [46]. The sharing is pre-/nonverbal and experiential, with body sensations and affects as essential components [32]. The primary experiences of competency, body mastery, and sensations are emergently grounded in this field.

Experiences of intersubjective regulation are achieved and developed through musical elements such as rhythm, melody, movement, and dynamic shifts [47,64]. In both expressive and receptive music therapy, shared intentions and affects do not need to be translated to language; they are cross-modal, involving different sensory modalities [32]. Attunement through musical activities in music therapy gives the experience of being able to interact and influence, and to sense one’s competency [32], which is one way to experience one’s self at best.

Vitality is a concept developed by Daniel Stern to describe the feeling of being alive, and in Active music therapy it is important for the music therapist to be aware of different forms of vitality in improvisation, sharing, and nonverbally communicating the experience of being in the music together [65].

The properties of music are powerful; music can regulate emotions, reduce stress levels, and strengthen the experience of belonging, attachment, and social bonds [23,26]. These aspects are central in music therapy treatment in the hospital setting [31]. The results in this study show the importance of offering music therapy in a secure setting for children and families in the vulnerable situation of undergoing HSCT.

The data collection method, the collaborative research interview, aimed to have the collaboration between the music therapist and child/parents in focus and learn from their feedback. Music therapy for the participants developed into a significant and helpful experience. In this experience, the relationship and collaboration with the music therapist through meeting and playing together became a significant ingredient in coping with and managing the treatment period at the hospital. These findings illuminated contributing factors in accordance with our previous findings of reduced heart rates four to eight hours after a music therapy session and increased levels of HRQoL after music therapy intervention. The longing for more sessions expressed by the participants can be understood as mirroring the importance of the developed relationship with the music therapist and musicking. The interruption of ordinary family life and experience of isolation were two of the main themes in a study of siblings of HSCT patients [15,17].

Due to research conditions, the framework in this special context was a predetermined number of sessions. The question about the dose or length of the therapeutic process is important, and clinical work requires flexibility in meeting the needs of the child. Our previous research showed that the doses in the randomized study were sufficient to obtain significant effects [43,44]. The results also point out the importance of diminished feelings of abandonment and, despite clinical challenges, facilitate completion of the therapeutic process as much as possible.

### 4.1. Limitations

This study focused on six families. Our results are based on their personal and unique experiences of music therapy in a special and exposed context. It should be mentioned that the group was heterogeneous in terms of age, disease severity, duration of illness, ethnicity, social affiliation, pretreatment, relapse risk, and complication risk. A limitation may be that the previous quantitative results and this study’s qualitative results on the six children are not merged. What can be perceived as strengths in the study were that all children were treated in the same hospital ward, received equivalent information, and met the same music therapist and the same interviewer.

### 4.2. Further Research

Our intervention primarily focused on sick children, although parents and siblings were welcome to attend. Earlier research and our study showed that the entire family is affected by HSCT and it would be desirable to develop and evaluate music therapy interventions that encompass the whole family when the child is undergoing HSCT. Additionally, the importance of dosing pre- and post-HSCT is an issue for further research.

## 5. Conclusions

The interview study documents that for the participants, music therapy developed into a significant and helpful experience. In this experience, the relationship and collaboration with the music therapist through meeting and playing together became a significant ingredient in coping with and managing the treatment period at the hospital.

## Appendix A

**Table A1:** Questions of the inquiry.

**Questions**
In the following, the questions of the inquiry are described with their underlying intention.
**How was it to be involved in music therapy?**
The focus of this question was the overall experience of the participants, child as well as parents and siblings, of being involved with music therapy. The focus was on the general experience of the entire music therapy process.
**How was it to play music in this way?**
The focus was on the phenomenological subjective experience of interacting and collaborating with the music therapist when musicking, to find the participants’ descriptions of experiencing the musicking in detailed and concrete ways. The musicking is assumed to contain/encompass collaborative and interactive regulation.
**What was the best/the worst?**
In this question, the focus was on finding and exploring even more specific moments in the interaction and collaboration, moments with both a positive and negative experiential flavor.
**Is there anything special you remember from when you played, sang, improvised?**
This question was connected to the previous one, and was intended to deepen the exploration of memories from the music therapy process. It is assumed that moments of interaction and collaboration that stand out in memory carry a certain affective quality or intensity. As such, they were assumed to contain potential for change.
**How was it to get music therapy during the HSCT/after the HSCT?**
It was important to evaluate the participants’ experience of being involved in music therapy both during and after HSCT, to understand how this affected the experience of music therapy.
**The therapist’s perspective: Ad hoc questions formulated when meeting the child and the parents.**
According to the assumptions behind the format of the collaborative interview, the opportunity to explore collaborative and interactive qualities of a certain therapeutic intervention is connected to the therapist’s subjective perspective of the collaboration. Therefore, this theme of inquiry was explored to access the memories and experiences of the participants. This is further discussed in the Discussion section of the paper. The last two questions were intended to facilitate reflection by the participants on the overall process from a meta-perspective.

**Table A2:** Excerpts from the analysis of interviews with six children and their parents after receiving music therapy. Participants included: A, post-HSCT; B, during HSCT; C, during HSCT; D, post-HSCT; E, during HSCT; F, post HSCT. Music therapist is abbreviated as T. Verbal testimonies are in italics and the interviewer’s memories of interactions are in ordinary text.

**1. How was it to be involved in music therapy?**
**1.1. Musicality was evoked**
Mother: *We noticed in A, A loves music*. (A)
Laughter during interview when listening to recordings from the MT sessions. (B)
Mother: *I am moved when C tells us this.* (C)
Mother: *When we came home, D wanted a guitar. D got more interested in music.* (D)
During the interview, participants shared memories through singing together. (E)
Mother: *I myself like music, we sing in church.* (F)
**1.2. Monotonous isolation was broken**
Mother: *The thing was that we got away from it, A could leave the room.* (A)
Child: *I got cheerful, happy when T came into my room*. (C)
Mother: *Asked after each session, when is next.* (D)
Father: *When T came, E wanted to leave E’s mom and that was fun for all of us.* (E)
**1.3. A “sense of normality” was evoked**
Mother: *A became revived, could come out—it was life in it.* (A)
Mother: *B had a positive experience, felt better, became less gloomy. B breathed more freely.* (B)
Mother: *After C sang together with T, C experienced it [the treatment] differently.* (C)
Mother: *all of us got happy, [sibling] too. Joy, happy, to feel alive.* (F)
**1.4. Child’s competencies were activated**
Mother: *A got a task and purpose.* (A)
During the interview, B played recordings of the songs B made together with T and expressed in head nods that it was familiar for B to sing songs and that it felt safe for B to sing. (B)
Mother: *It was good that B could decide/suggest.* (B)
Child: *When I sang with T, I forgot the pain.* (C)
**1.5. Music therapy offered pain diversion**
Mother: *Became easier [for B] to breathe. It affected the senses.* (B)
Child: *When I met T in MT I forgot the pain.* (C)
Father: *E became happy, and at the same time very tired*. (E)
**1.6. Music therapy evoked regulation of different kinds of affects**
Mother about B: *Easier to breathe, affected B’s mind. It was like T captured when B was enclosed a clam like*. (B)
Child: *I felt joy when T entered my room. I love music.* (C)
Mother: *We felt safe and happy after each MT session.* (D)
Father: *When they have fun, I also feel happy.* Mother: *It helped me to see them happy, [because] it was very hard.* (E)
Mother: *F became happier, F smiled at once. We all became happy.* (F)
***2.* How was it to play music in this way?**
**2.1. Music therapy activated bodily sensations and affects**
Mother: *A felt happy, A recognized, longed [for MT]. It was fun. How it felt in the body.* (A)
B smiled and nodded during interview. Father confirmed that B felt safe. (B)
Mother: *D got more interested, happy after each meeting. D shows during interview: “aha!” you can do this!* (D)
Father: *It was great fun, E became happy. It was fun for all of us.* (E)
Mother: *That half hour, was very fun, F liked, danced.* (F)
**2.2. Emergence of interactive regulation**
Father and B conveyed that it suited B, it was familiar to B to play and sing, B knew many songs before. Father expressed that the familiar became safe for B. (B)
Child: *I noticed that the pain was affected, I could also listen.* (C)
During interview, D showed a video on the iPhone from one MT session, and conveyed that it was fun and important. (D)
Mother: *When T learned a song from our native country*, *F started to dance, it evoked something*. (F)
**3. What was the best/the worst?**
**4. Is there anything special you remember from when you played, sang, improvised?**
**3, 4 Evoked positive experiences also contrasted with the longing for more sessions**
Mother: *It was fun to play together. T always took her time. We felt recognized. We felt remembered, one is a human being. One feels warmth in heart*. (A)
Father: *I remember when we wrote songs, it was nice interplay. B revived, felt confident in her/him self. It was laughter, hilariousness*. (B)
Child: *Remember once we could not do anything [play, due to too much pain], did not feel good when she left. Once, in the shower the pain went down when I started to sing.* (C)
Mother: *D felt sad the last time. Then D got more aroused. It was too short*. (D)
Father: *Noticed that E became happier, that E and E’s sibling played together and that sibling helped E to play. Sibling waited for T all the time. Every second day would have been better.* (E)
Mother: *Once, new energy when Fs uncle came. Relatives [from…] took part.* (F)
**5. How was it to get music therapy during the HSCT/after HSCT?**
**5. The experienced period was appreciated**
Mother: *We found it was best as it was.* (A)
Father: *Better during HSCT, a break in the room.* Mother: *100% best—if not, we had not created/written those songs*! (B)
Child: *It was best during HSCT, it was when I needed it.* (C)
Mother: *It was best after HSCT, D had more strength.* (D)
Mother: *Perhaps better during HSCT*. Mother expressed it would have felt better when the child was small. *We are one.* (F)
**6. The therapist’s perspective: Ad hoc questions formulated when meeting the child and parents.**
**6. Mutuality and sharing in the collaboration**
Recollects A’s musicality with many songs of A’s own. Mother and Father met A, A engaged them. (A)
B does not speak but shows a positive face and nods. B and Father convey that it suited B very well, it was familiar to B to sing, B knew many songs before. Father thinks that the familiar became safe for B. (B)
Recollects C’s curiosity, and that they made music together. Remembers that C had pain, and now asks how it was for C to play in spite of pain. (C)
Recollects the family as four strongly tight together. A lot of energy. Sibling, when E was tired, played, and it was good for E. Sibling helped E to become interested, helped the little sibling. T sometimes felt she interrupted. E was in the music together with E’s sibling. They really interacted, interplayed. (E)
T and F meet each other through playing together for a while, since F immediately turns towards the instruments that F recognizes from the MT sessions. T remembers it being simple, F’s positive expectancies and desire to be with the music. T also remembers that mother supported through her personality and way of engaging. It was helpful to the T. (F)
When T and D meet, they reconnect nonverbally and remain in interactive musicking during the interview, without any verbal dialogue. The meeting seemed like a continuation of their former process. D, through verbal statements from the mother, conveyed that the music therapy was too short. (D)

**Table A3:** Each question is followed by analyzed assumptions.

**1. How was it to be involved in music therapy?**
1.1. The entire experience evoked musicality, both in the here and now and as a recollecting process of former experiences of music. 1.2. Music therapy was experienced as a break in the monotonous isolation necessary for the treatment. This was assumed to contribute to the participant’s capacity to preserve a connection to life outside the hospital. 1.3. The music therapy sessions evoked a sense of “normality” connected to the participant’s normal life and a sense of being in life, being alive. 1.4. Music therapy evoked participants’ own competencies, like former musical activities and a general sensorimotor competency. 1.5. Music therapy evoked diversion from pain, and presumably also from fear. 1.6. Music therapy activated affects: curiosity, joy, liveliness, and energy arousal.
**2. How was it to play music in this way?**
2.1. Music therapy activated bodily sensations of joy, curiosity, and energy arousal, which is a deeper level of experiencing and regulating one’s affects. 2.2. Music therapy generated and evoked memories of experiences of competency, self at best, self-assertion. The act of learning and playing songs was connected to the ability to express a sense of self. In the domain of interaction and regulation, the sharing and regulating with the therapist, parents, and siblings generated experiences of recognition and positive affects in meeting and interactive regulation. It is assumed that it also generated memories of interactive regulation and self-regulation. Sensorimotor, tactile, auditory, and visual experiences of handling instruments, singing, and making body movements were assumed to evoke implicit memories of competency, self-regulation, and interactive regulation of arousal levels and affects. These, in turn, amplified experiences of safety and trust, affirmation and confirmation. In the following, responses to two questions are depicted together, since the answers conveyed that they were connected according the experiences of the participants. The question about what was the best/the worst (question 3) seemed to be equal to memories of “special moments” (question 4). The questions were:
**3. What was the best/the worst?**
**4. Is there anything special you remember from when you played, sang, improvised?**
The responses illuminate highlights of being recognized and confirmed. Sometimes tiredness and pain were in the way. The experience often evoked a longing for more sessions. The evoked positive experiences also contrasted with the longing for and shortage of sessions. Some participants remembered specific songs they learned or composed. Others remembered moments of change, such as when the therapist left or ended a session, which triggered feelings of sadness or worry.
**5. How was it to get music therapy during the HSCT/after the HSCT?**
All participants appreciated the period they experienced. Some emphasized that they appreciated music therapy both during and after HSCT.
**6. The therapist’s perspective: Ad hoc questions formulated when meeting the child and parents.**
The therapist’s memories of each participant and her/his family were taken as a point of departure in each interview. Through the recollecting process in the meeting, six personalized narratives emerged. The common ingredient in each meeting appeared to be how the child, during music therapy, reconnected to his/her relationship with music. The parents were often part of the process, present in different ways depending on the age of the child. Some were just present in the room and some actively took part in the musical interaction. From the perspective of the therapist, they were all felt to be facilitators of the process. The therapist remembered several moments of meeting and of interaction characterized by positive emotions such as joy, laughter, and curiosity. She also remembered how the children’s specific sense of self, or character, was displayed during music therapy. In the process of recollection, the therapist often described her sense of mutuality or reciprocity in the collaboration, and she also expressed how she learned from the meetings. One meeting, with participant D, induced a kind of continuation of the therapeutic process as D stayed in a nonverbal interaction, playing on different instruments with the therapist during the whole interview.

## References

[1] Miano M., Labopin M., Hartmann O., Angelucci E., Cornish J., Gluckman E., Locatelli F., Fischer A., Egeler R.M., Or R. (2007). Haematopoietic stem cell transplantation trends in children over the last three decades: A survey by the paediatric diseases working party of the european group for blood and marrow transplantation. Bone Marrow Transpl..

[2] Remberger M., Ackefors M., Berglund S., Blennow O., Dahllöf G., Dlugosz A., Garming -Legert K., Gertow J., Gustafsson B., Hassan M. (2011). Improved survival after allogeneic hematopoietic stem cell transplantation in recent years. A single-center study. Biol. Blood Marrow Transpl..

[3] Tanzi E.M. (2011). Health-related quality of life of hematopoietic stem cell transplant childhood survivors: State of the science. J. Pediatr. Oncol. Nurs..

[4] Rodgers C., Wills-Bagnato P., Sloane R., Hockenberry M. (2015). Health-related quality of life among children and adolescents during hematopoietic stem cell transplant recovery. J. Pediatr. Oncol. Nurs..

[5] Tremolada M., Bonichini S., Pillon M., Messina C., Carli M. (2009). Quality of life and psychosocial sequelae in children undergoing hematopoietic stem-cell transplantation: A review. Pediatr. Transpl..

[6] Rodday A.M., Terrin N., Leslie L.K., Graham R.J., Parsons S.K. (2017). Understanding the relationship between child health-related quality of life and parent emotional functioning in pediatric hematopoietic stem cell transplant. J. Pediatr. Psychol..

[7] Tremolada M., Bonichini S., Basso G., Pillon M. (2016). Post-traumatic stress symptoms and post-traumatic growth in 223 childhood cancer survivors: Predictive risk factors. Front. Psychol..

[8] Stuber M.L., Nader K., Yasuda P., Pynoos R.S., Cohen S. (1991). Stress responses after pediatric bone marrow transplantation: Preliminary results of a prospective longitudinal study. J. Am. Acad. Child Adolesc. Psychiatry.

[9] Buchbinder D., Kelly D.L., Duarte R.F., Auletta J.J., Bhatt N., Byrne M., DeFilipp Z., Gabriel M., Mahindra A., Norkin M. (2018). Neurocognitive dysfunction in hematopoietic cell transplant recipients: Expert review from the late effects and quality of life working committee of the cibmtr and complications and quality of life working party of the EBMT. Bone Marrow Transpl..

[10] Cozolino L.J. (2010). The Neuroscience of Psychotherapy: Healing the Social Brain.

[11] (2013). Diagnostic and Statistical Manual of Mental Disorders: DSM-5.

[12] Siegel D.J. (1999). The Developing Mind: Toward a Neurobiology of Interpersonal Experience.

[13] Phipps S., Dunavant M., Lensing S., Rai S.N. (2005). Psychosocial predictors of distress in parents of children undergoing stem cell or bone marrow transplantation. J. Pediatr. Psychol..

[14] Jobe-Shields L., Alderfer M.A., Barrera M., Vannatta K., Currier J.M., Phipps S. (2009). Parental depression and family environment predict distress in children before stem cell transplantation. J. Dev. Behav. Pediatr..

[15] Packman W., Weber S., Wallace J., Bugescu N. (2010). Psychological effects of hematopoietic sct on pediatric patients, siblings and parents: A review. Bone Marrow Transpl..

[16] Switzer G.E., Bruce J., Kiefer D.M., Kobusingye H., Drexler R., Besser R.M., Confer D.L., Horowitz M.M., King R.J., Shaw B.E. (2016). Health-related quality of life among pediatric hematopoietic stem cell donors. J. Pediatr..

[17] Hutt D., Nehari M., Munitz-Shenkar D., Alkalay Y., Toren A., Bielorai B. (2015). Hematopoietic stem cell donation: Psychological perspectives of pediatric sibling donors and their parents. Bone Marrow Transpl..

[18] Riva R., Forinder U., Arvidson J., Mellgren K., Toporski J., Winiarski J., Norberg A.L. (2014). Patterns of psychological responses in parents of children that underwent stem cell transplantation. Psychooncology.

[19] Wills T.A., Simons J.S., Sussman S., Knight R. (2016). Emotional self-control and dysregulation: A dual-process analysis of pathways to externalizing/internalizing symptomatology and positive well-being in younger adolescents. Drug Alcohol Depend..

[20] Bowlby J., Wiking P., Risholm Mothander P. (2010). En Trygg Bas: Kliniska Tillämpningar av Anknytningsteorin.

[21] Bergsten K. (2015). Affektfokuserad Psykodynamisk Terapi: Teori, Empiri och Praktik.

[22] Stern D.N. (2000). The Interpersonal World of the Infant: A View from Psychoanalysis and Developmental Psychology.

[23] Chanda M.L., Levitin D.J. (2013). The neurochemistry of music. Trends Cogn. Sci..

[24] Finn S., Fancourt D. (2018). The biological impact of listening to music in clinical and nonclinical settings: A systematic review. Prog. Brain Res..

[25] Fancourt D., Ockelford A., Belai A. (2014). The psychoneuroimmunological effects of music: A systematic review and a new model. Brain Behav. Immun..

[26] Moore K.S. (2013). A systematic review on the neural effects of music on emotion regulation: Implications for music therapy practice. J. Music.

[27] Theorell T. (2014). Psychological Health Effects of Musical Experiences [Elektronisk Resurs]: Theories, Studies and Reflections in Music Health Science.

[28] Juslin P.N., Sloboda J.A. (2010). Handbook of Music and Emotion: Theory, Research, and Applications.

[29] Sena Moore K., Hanson-Abromeit D. (2015). Theory-guided therapeutic function of music to facilitate emotion regulation development in preschool-aged children. Front. Hum. Neurosci..

[30] Malloch S., Trevarthen C. (2008). Communicative Musicality: Exploring the Basis of Human Companionship.

[31] Dun B., Bradt J. (2012). Children with cancer. Guidelines for Music Therapy Practice in Pediatric Care [Elektronisk Resurs].

[32] Trondalen G. (2016). Intersubjectivity and Development as a dialogical continuum. Relational Music Therapy: An Intersubjective Perspective.

[33] Birnbaum J.C. (2014). Intersubjectivity and nordoff-robbins music therapy. Music Ther. Perspect..

[34] Stige B. (2002). Culture-Centered Music Therapy.

[35] Small C. (1999). Musicking—The meanings of performing and listening. A lecture. Music Educ. Res..

[36] DeNora T. (2005). The pebble in the pond: Musicing, therapy, community. Nord. J. Music Ther..

[37] Robb S.L., Clair A.A., Watanabe M., Monahan P.O., Azzouz F., Stouffer J.W., Ebberts A., Darsie E., Whitmer C., Walker J. (2008). A non-randomized [corrected] controlled trial of the active music engagement (ame) intervention on children with cancer. Psychooncology.

[38] Nguyen T.N., Nilsson S., Hellstrom A.L., Bengtson A. (2010). Music therapy to reduce pain and anxiety in children with cancer undergoing lumbar puncture: A randomized clinical trial. J. Pediatr. Oncol. Nurs..

[39] Barrera M.E., Rykov M.H., Doyle S.L. (2002). The effects of interactive music therapy on hospitalized children with cancer: A pilot study. Psychooncology.

[40] Tucquet B., Leung M. (2014). Music therapy services in pediatric oncology: A national clinical practice review. J. Pediatr. Oncol. Nurs..

[41] Robb S.L., Ebberts A.G. (2003). Songwriting and digital video production interventions for pediatric patients undergoing bone marrow transplantation, part I: An analysis of depression and anxiety levels according to phase of treatment. J. Pediatr. Oncol. Nurs..

[42] Robb S.L., Burns D.S., Stegenga K.A., Haut P.R., Monahan P.O., Meza J., Stump T.E., Cherven B.O., Docherty S.L., Hendricks-Ferguson V.L. (2014). Randomized clinical trial of therapeutic music video intervention for resilience outcomes in adolescents/young adults undergoing hematopoietic stem cell transplant: A report from the children’s oncology group. Cancer.

[43] Uggla L., Bonde L.O., Svahn B.M., Remberger M., Wrangsjö B., Gustafsson B. (2016). Music therapy can lower the heart rates of severely sick children. Acta Paediatr..

[44] Uggla L., Bonde L.O., Hammar U., Wrangsjo B., Gustafsson B. (2018). Music therapy supported the health-related quality of life for children undergoing haematopoietic stem cell transplants. Acta Paediatr..

[45] Trondalen G., MacDonald R.A.R., Kreutz G., Mitchell L. (2012). Bonde LO. Music Therapy: Models and Interventions. Music, Health, and Wellbeing.

[46] Fosha D., Siegel D.J., Solomon M.F. (2009). The Healing Power of Emotion: Affective Neuroscience, Development, Clinical Practice.

[47] Bonde L.O.R. (2014). Musikterapi Teori Uddannelse Praksis Forskning en Håndbog om Musikterapi i Danmark.

[48] Murphy K., Wheeler B.L. (2016). Music Therapy Research [Elektronisk Resurs].

[49] Andersen T., Weine B., Hopstadius K. (2011). Reflekterande Processer: Samtal och Samtal om Samtalen.

[50] Blom K.M. (2014). Experiences of Transcendence and the Process of Surrender in Guided Imagery and Music (GIM). Ph.D. Thesis.

[51] Kvale S., Brinkmann S. (2009). Interviews: Learning the Craft of Qualitative Research Interviewing.

[52] Andersen T. (1997). Researching client-therapist relationships: A collaborative study for informing therapy. J. Syst. Ther..

[53] Blom K.M. (2006). Samspela, samtala, samforska—Om ömsesidiga processer I terapi och forskning. Fokus På Familien.

[54] Fosha D. (2000). The Transforming Power of Affect: A Model for Accelerated Change.

[55] Sander L.W. (2002). Thinking differently principles of process in living systems and the specificity of being known. Psychoanal. Dialogues.

[56] (2010). Change in Psychotherapy: A Unifying Paradigm.

[57] Tronick E. (2007). The Neurobehavioral and Social Emotional Development of Infants and Children.

[58] Schore A.N. (2003). Affect Regulation & the Repair of the Self.

[59] Steele K., Boon S., van der Hart O. (2017). Treating Trauma-Related Dissociation: A Practical, Integrative Approach.

[60] Bonde L.O., Blom K.M. (2016). Music listening and the experience of surrender. Cultural Psychology of Musical Experience.

[61] Fukunishi I., Tsuruta T. (2001). Alexithymic characteristics in children with refractory hematological diseases. Psychosomatics.

[62] Phipps S., Klosky J.L., Long A., Hudson M.M., Huang Q., Zhang H., Noll R.B. (2014). Posttraumatic stress and psychological growth in children with cancer: Has the traumatic impact of cancer been overestimated?. J. Clin. Oncol..

[63] Mårtenson Blom K., Wrangsjö B. (2013). Intersubjektivitet: Det Mellanmänskliga i Vård och Vardag.

[64] Bjørkvold J.-R., Nilsson L., Schollin-Borg K. (2005). Den Musiska Människan.

[65] Stern D.N. (2010). Forms of Vitality: Exploring Dynamic Experience in Psychology, the Arts, Psychotherapy, and Development.

